# Serum agonistic autoantibodies against type-1 angiotensin II receptor titer in patients with epithelial ovarian cancer: a potential role in tumor cell migration and angiogenesis

**DOI:** 10.1186/1757-2215-6-22

**Published:** 2013-04-05

**Authors:** Li Song, Su-Li Zhang, Ke-Hua Bai, Jie Yang, Hai-Yan Xiong, Xiao Li, Teng Liu, Hui-Rong Liu

**Affiliations:** 1 Department of Pathophysiology, Capital Medical University, Beijing, P.R. China

**Keywords:** Angiotensin II type I receptor, Autoantibodies against type-1 angiotensin II receptor, Angiogenesis, Epithelial ovarian cancer

## Abstract

**Background:**

Although agonistic autoantibodies against type-1 angiotensin-II receptor (AT_1_-AA) are frequently detected in women with preeclampsia, the clinical significance of AT_1_-AA in association with epithelial ovarian cancer (EOC) has not been identified.

**Methods:**

In an attempt to clarify this issue, we measured serum AT_1_-AA titer from EOC patients (n = 89) and healthy normal subjects (n = 55), correlated AT_1_-AA titer with EOC stage and grade, and demonstrated the effects of purified AT_1_-AA on migration of ovarian cancer cells and angiogenesis of chick embryo chorioallantoic membrane.

**Results:**

We found that the AT_1_-AA titer was significantly higher in EOC patients compared with healthy control subjects (1.77 ± 0.28 vs. 0.35 ± 0.05, P < 0.01). The positive rate was averaged by 72.1±2.5% in EOC patients and 15.5 ±1.5% in control (P < 0.01). Increased AT_1_-AA titer in EOC patients was associated with advanced stages and grades of EOC, and positively correlated with level of vascular endothelial growth factor (r = 0.855, P < 0.01). Furthermore, AT_1_-AA directly stimulated migration of ovarian cancer cells and enhanced microvascular density of chick embryo chorioallantoic membrane. These AT_1_-AA-mediated effects were significantly blocked either by an autoantibody-neutralizing peptide or an angiotensin II type I receptor antagonist, losartan.

**Conclusion:**

Taken together, we found that a higher serum AT_1_-AA titer may be associated with advanced progression of EOC in patients and play an important role in development of EOC by promoting cancer cell migration and angiogenesis. These findings implicate that AT_1_-AA might be selected as a detectable biomarker and potential therapeutic target in diagnosis and treatment of EOC patients.

## Background

Epithelial ovarian cancer (EOC) is the sixth most common cancer and the seventh cause of death worldwide among women who develop gynecological cancer [1], with the estimated 22,280 new cases and 15,500 deaths in the United States in 2012. The vast majority of EOC patients are usually diagnosed with advanced stages due to the lack of adequate early screening tests and early specific symptoms during development of EOC [2]. The standard treatment for advanced EOC patients includes debulking surgery followed by platinum–taxane based chemotherapy. These patients, however, are also at great risk of recurrence and emerging drug resistance with a more than 70% of relapse rate and a mean 18 months of progression-free survival period [2,3]. Therefore, understanding the pathogenesis of EOC and identifying early detectable biomarkers are essential to improve overall survival rate in advanced EOC patient.

It has well been defined that angiotensin II (Ang II) derived from the activated renin-angiotensin system plays a key role in the regulation of cardiovascular homeostasis through its two receptors: Ang II type 1 (AT_1_) and type 2 (AT_2_) receptors, which maintain arterial blood pressure, fluid and electrolyte homeostasis. Through the AT_2_ receptor, Ang II evokes vasodilatation, sodium excretion and blood pressure reduction, and thereby counteracts the effects of AT_1_ receptor [4]. However, increasing evidence suggests that Ang II is also involved in tumor cell migration/invasion, angiogenesis and metastasis through AT_1_ receptor during the tumor development [5-7]. In patient with EOC, it has previously been reported that Ang II enhances vascular endothelial growth factor (VEGF) secretion, angiogenesis and tumor cell invasion via up-regulating G-protein-coupled AT_1_ receptor; importantly, angiogenesis and peritoneal dissemination of the EOC can selectively be blocked using AT_1_ receptor antagonist [6,8]. Therefore, considerable effort has been placed on the development of Ang II blockade therapy as a new strategy for EOC treatment.

Recent studies have demonstrated that agonistic autoantibodies against type-1 angiotensin II receptor (AT_1_-AA) detected in preeclampsia induces significant placental trophoblast invasion [9], suggesting that AT_1_-AA is one of the potential causative factors in development of preeclampsia. We have previously reported that AT_1_-AA constricts human fetoplacental blood vessels and restricts fetal perfusion through activating Ang II AT_1_ receptor [10]. Although animals studies have shown that activation of AT_1_-AA is associated with elevation of intracellular Ca^2+^ in vascular smooth muscle cells [11], stimulation of placental and vascular NADPH oxidase [12] and activation of NF-κB [13], all of which may cause inflammation and contribute to pathogenesis of preeclampsia via AT_1_-AA, there is less specific data to show whether AT_1_-AA is elevated in patient with EOC and correlated with the advanced progression of EOC. Therefore, in the current study, we examined the serum AT_1_-AA titer in EOC patients and determined whether change in AT_1_-AA level is associated with malignant grades and angiogenic factor, VEGF. Using AT_1_-AA purified from EOC patients, we demonstrated the effects of AT_1_-AA on migration of ovarian cancer cells and microvascular density of chick embryo chorioallantoic membrane. Furthermore, we investigated whether the AT_1_-AA-elicited biological effects could be suppressed by autoantibody-neutralizing AT_1_-AA peptide, and whether cell migration and angiogenesis stimulated by AT_1_-AA could be blocked by Ang II AT_1_ receptor antagonist.

## Methods

### Patients

The study included 89 malignant EOC patients who were diagnosed and operated in the third hospital of Capital Medical University during the period of 05/2010 to 04/2012. Cases were chosen based on the histological grades and clinical stages of EOC patients according to the International Federation of Gynecology and Obstetrics (FIGO) criteria. The healthy control subjects (n = 55) were enrolled from laparoscopy-negative cases on the clinical assessment at the same hospital. No significant difference in age was found between these two groups. The consent form was signed by all patients and the research protocol was approved by the Institutional Committee for the Protection of Human Subjects of Capital Medical University. Cases were excluded if patients were associated with 1) autoimmune diseases and endocrinal diseases; 2) complications derived from other different organ systems; 3) immune deficiencies diseases; 4) significant gastrointestinal diseases. All clinical and laboratory data were recorded. Serum samples were collected from the patients in both groups and stored at −80°C until use.

### Measurement of AT_1_-AA titer and VEGF by enzyme-linked immunosorbent assay (ELISA)

The serum AT_1_-AA level in patients was detected by ELISA as we reported previously [10]. Briefly, 96-well microtiter plates were coated with 1 μg/ml AT_1_R-ECII peptide synthesized from patients (GL Biochem Ltd, Shanghai, China) and incubated overnight at 4°C. After washing the plates with PBS three times, 50 μl serum samples were added to the plates and incubated at 37°C for 1 h. The biotinylated goat anti-human IgG antibody (1:3,000, Zhongshan Inc., Beijing, China) or streptavidin-peroxidase conjugate (1:2,000 Vector, CA, USA) was then incubated separately at 37°C for 1 h during washings. Finally, 2, 2-azino-di (3-ethylbenzothiazoline) sulphonic acid (ABTS)-H_2_O_2_ (Roche, Basel, Switzerland) substrate buffer was applied for a half hour before reading. The optical densities (OD) from these plates were measured at 405 nm in a plate reader (Molecular Devices Corp, CA, USA). The AT_1_-AA titer was expressed as the ratio of positive/negative (P/N), i.e., (the OD of specimen - the OD of blank control) / (the OD of negative control - the OD of blank control). The positivity of the serum sample to AT_1_-AA was defined as P/N ≥ 2.1, while the negativity was defined as P/N ≤ 1.5. All assays were performed in duplicate. Commercially accessible ELISA kit (DaKeWe Biotechnological Corp, Shengzheng, China) were used to determine the patients’ serum VEGF level according to the manufacturer’s instructions. VEGF concentration was expressed as ng/L and the assays were performed in duplicate.

### AT_1_-AA peptide synthesis

AT_1_-AA peptide fragments equivalent to the sequence of human anti-AT_1_ receptor antibody (AT_1_R-ECII, 165–191, I-H-R-N-V-F-F-I-I-N-T-N-I-T-V-C-A-F-H-Y-E-S-Q-N-S-T-L) was synthesized by solid-phase peptide synthesis method (GL Biochem Ltd, Shanghai, China). The purity of synthetic peptide was confirmed with a high pressure liquid chromatography as we reported previously [10].

### Purification of the immunoglobulin G fraction

The total immunoglobulin G was isolated from serum samples with AT_1_-AA positive EOC patients or AT_1_-AA negative healthy normal subjects by Mab Trap Kit (Amersham, NJ, USA). The purities of extractions were assessed by sodium dodecylsulfonate–polyacrylate gel electrophoresis (SDS–PAGE) as we reported previously [10].

### Cell lines and cell migration assay

Human ovarian cancer cells (OVCAR3) were purchased from the Cancer Hospital of Chinese Academy of Medical Sciences, Beijing, China and maintained in DMEM supplemented with 10% FBS, 2 mM l-glutamine, 100 units/ml penicillin and 100 μg/ml streptomycin. For all experiments, cells were detached with 0.25% trypsin and 0.02% EDTA and washed once in complete medium before use. Migration assay was conducted according to the manufacture’s recommended protocol (BD Biosciences, New Jersey, USA). Briefly, OVCAR3 at 5 × 10^4^ concentration were suspended in 300 μl of serum free media in the upper chamber with pre-coated filters (6.5 mm in diameter and 8 μm pore-size) with or without AT_1_-AA, Ang II, AT_1_R-ECII or Ang II AT_1_ receptor antagonist, losartan. Bottom chambers were filled with medium containing 10% FBS as a chemoattractant. After cells were allowed to seed on the chambers for 24 h at 37°C, cells on the upper chamber and migrated cells at the bottom chamber were wiped with a cotton swab and then mixed with staining solution containing 0.125% coomassie blue in a mixture of methanol, acetic acid and water in a ratio of 45:10:45. The results were visualized under an inverted microscope from 5 randomized high power fields (x400). Results were calculated from the average of 3 separate assays conducted in triplicate.

### Visualization **of microvascular density in chick embryo chorioallantoic membrane (CAM)**

Fertilized white leghorn chicken eggs were received at day 0 and incubated for 3 days at 37°C with constant humidity. On day 3, eggs were rinsed with 70% ethanol and a square window (0.5 cm^2^) was made with a pair of sterile scissor and cut away a circle of shell, thus exposing the underlying membrane (the chorioallantois). After the eggs (n = 8/each group) were treated with saline, AT_1_-AA, Ang II, AT_1_R-ECII or losartan, respectively for 30 min, the window was sealed with transparent tape and the eggs returned to the incubator at 90% relative humidity without turning. After 72 h of incubation, the CAM was fixed using 3.7% formaldehyde for 15 min, cut 3 cm^2^ from the center and mounted on the slides for observation. The angiogenic results were visualized on an inverted microscope from 5 randomized fields. For each experiment, the staggered images were digitized and results were calculated as a mean of microvascular density per high power field (x 400).

### Statistical analysis

All data were calculated as mean ± SE. Statistical analysis was performed with SPSS 15.0 software. The positive rates in the two groups were compared with chi-square test. The *t*-test was applied for comparing two independent sample means, and the one-way ANOVA was used for comparing means of more than two samples. P < 0.05 was considered to be statistically significant.

## Results

### Clinical characteristics presented in EOC patients

Patient characteristics, stage and grade are shown in Table 1. The mean age of the EOC at primary diagnosis was 50.4±11 years and the mean history of the EOC was 7.4 ± 6 years. The mean age of the EOC at menarche was 15±2, and at menopause was 47±4. The FIGO stage of EOC patients was classified as follows: I: 6%; II: 56%; III: 23%; IV: 4%. Most EOC patients were at the grade III (61%). Fifty-four patients (28%) had ascites whereas 31% patients had no such complication.

**Table 1:** Patient characteristics

**Characteristic**	**Number**
Age at diagnosis	51 + 8.2
Weight (kg)	62 + 8.9
BMI (kg/m2)	25 + 4.1
Age at menarche	15 + 2.0
Age at menopause	47 + 4.2
Stage	
I	6 (6.7%)
II	56 (62.9%)
III	23 (25.8%)
IV	4 (4.4%)
Grade	
G1	20 (22.4%)
G2	27 (30.3%)
G3	42 (85.7%)
Residual tumor	
≤2 cm	18 (20.2%)
>2 cm	71 (79.7%)
Ascites	
No	31 (34.8%)
Yes	58 (65.1%)
Diabetes Status	
No	30 (33.7%)
Yes	59 (66.2%)

Values are mean ± SE; others represent the percentage of total patients.

### Clinical significance of AT_1_-AA titer in EOC patients

**Figure 1:**
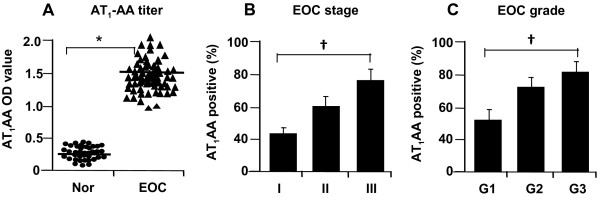
**Level of AT**_**1**_**-AA in EOC patients.** Relative to the healthy normal subjects, AT_1_-AA titer was significantly increased in EOC patients (**A**). The number of AT_1_-AA positive patients was associated with clinical FIGO stage (**B**) and grade (**C**). Values are mean ± SE. *p < 0.05 vs. the normal subjects (Nor); **†** P < 0.05 vs. the stage I and grade I, respectively.

The serum AT_1_-AA titer in EOC patients and healthy control subjects was measured by ELISA. As shown in Figure 1A, the serum AT_1_-AA titer was significantly increased from 0.35 ± 0.05 in healthy normal subjects to 1.77 ± 0.28 in EOC patients (P < 0.01). The average positive rate of AT_1_-AA in EOC patients was significantly higher than that in healthy normal subjects (72.1%±2.5% vs. 15.5%±1.9%, P < 0.01). The correlation of serum AT_1_-AA with clinicopathological outcomes was analyzed in EOC patients. As shown in Figure 1B, the number of AT_1_-AA positive patients was increased with clinical FIGO stage: 45% in stage 1, 61.5% in stage II and 72.8% in advanced stage III. Moreover, the AT_1_-AA titer was also significantly higher in patients with an advanced grade (Figure 1C): 61.7% in grade 1, 72.7% in grade II and 80.1% in grade 3. These results indicated that AT_1_-AA level increases with progression of EOC stage and grade.

### Correlation between serum AT_1_-AA titer and VEGF

**Figure 2:**
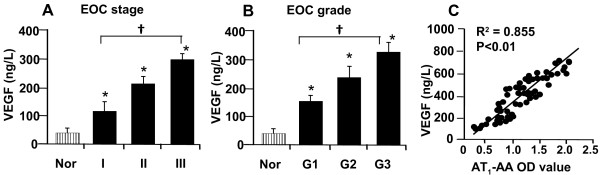
**VEGF level in EOC patients and healthy normal subjects.** Increased level of VEGF was detected in advanced stage (**A**) and grade (**B**). Scatter plots showed a positive linear correlation between VEGF level and AT_1_-AA titer in EOC patients (**C**). Values are mean ± SE. *p < 0.05 vs. the normal subjects (Nor); **†** P < 0.05 vs. the stage I and grade I, respectively.

To determine whether serum AT_1_-AA titer is associated with angiogenesis of the tumor, we examined the serum level of VEGF by ELISA in the same series of EOC patients. As shown in Figure 2A and 2B, VEGF level was significantly increased in patients with advanced FIGO stage and grade (i.e., at level III) compared with those in an early FIGO stage and grade (i.e., at level I). Positive linear correlation among the serum AT_1_-AA level and VEGF was detected, (Figure 2C, r^2^ = 0.855, p < 0.01), suggesting that AT_1_-AA may play a role in angiogenesis during development of EOC through enhancing VEGF expression.

### Effect of AT_1_-AA on migration of ovarian cancer cells

**Figure 3:**
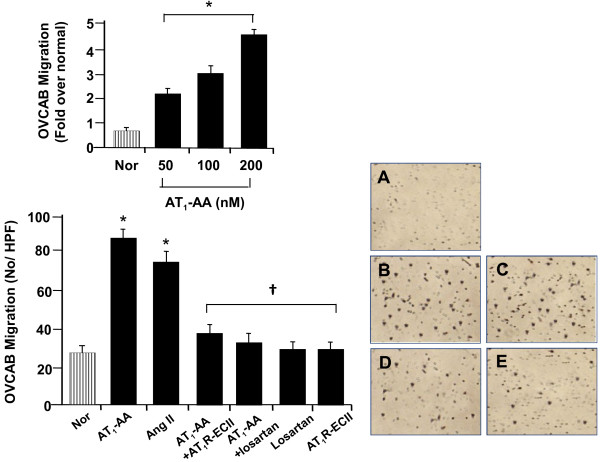
**Effect of AT**_**1**_**-AA on migration of OVCAR3 cells.** AT_1_-AA increased migration in a dose-dependent manner (top panel). Relative to the control (**A**), cell migration by AT_1_-AA was significantly enhanced (**B**), which is comparable to the level found in Ang II (**C**). Enhancement of migration by AT_1_-AA was blocked either by AT_1_-AA-ECII (**D**) or losartan (**E**). Values are mean ± SE. n = 3 for each group. *p < 0.05 vs. saline control (Nor); †P < 0.05 vs. AT_1_-AA and Ang II. HPF = high power field (x 400).

OVCAR3 cells derived from the progressive adenocarcinoma of the ovary were used in this study. Migration of OVCAR3 cells stimulated by adding AT_1_-AA was enhanced in a dose-dependent manner. As shown in the top panel of Figure 3, cell migration rates were consequently increased relative to the control when cells were treated with different dose of AT_1_-AA (50, 100, 200 nM) for 24 h. To demonstrate the potency of AT_1_-AA in stimulation of cell migration by activating angiotensin AT_1_ receptor, OVCAR3 cells were treated either with exogenous AT_1_-AA (100 nM) or Ang II (100 nM), respectively before subjecting to cell migration. As shown at the bottom panel of Figure 3, both AT_1_-AA and Ang II produced a comparable level in cell migration. Stimulation by AT_1_-AA (100 nM) on cell migration was completely blocked either by the AT_1_R-ECII (100 nM) or by the selective Ang II AT_1_ receptor antagonist, losartan (300 nM), suggesting that AT_1_-AA has direct stimulating effect on tumor cell migration and enhancement of OVCAR3 cell migration by AT_1_-AA is mediated by Ang II AT_1_ receptor. Addition of losartan or AT_1_R-EC II alone had no effect on migration of OVCAR3 cells.

### Effect of AT_1_-AA on angiogenesis of the CAM

**Figure 4:**
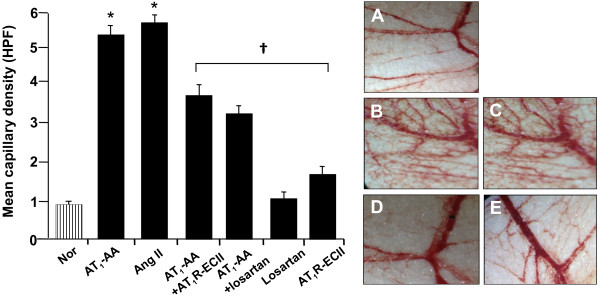
**Effect of AT**_**1**_**-AA on angiogenesis of the CAM.** Relative to the saline control (**A**), AT_1_-AA significantly increased microvascular density of the CAM (**B**) to a comparable level as did by Ang II (**C**). Enhancement of microvascular density by AT_1_-AA was blocked either by AT_1_-AA-ECII (**D**) or Ang II AT_1_ receptor antagonist, losartan (**E**). Values are mean ± SE. n = 6 for each group. *p < 0.05 vs. saline control (Nor); †P < 0.05 vs. AT_1_-AA and Ang II. HPF = high power field (x400).

AT_1_-AA administration caused a significant increase in microvascular density in the CAM. Figure 4 shows the representative photographs of AT_1_-AA-treated and saline control CAM. Quantitatively, in each of the six experiments, the microvascular density of the CAM treated with AT_1_-AA (100 nM) was increased by 60-70% compared with saline control. Addition of Ang II (100 nM) also increased the microvascular density of the CAM to a comparable level as that found in the AT_1_-AA treated CAM. Enhancement in the microvascular density by AT_1_-AA was significantly blocked either by AT_1_R-ECII (100 nM) or losartan (300 nM), suggesting a role of AT_1_-AA in angiogenesis through stimulating Ang II AT_1_ receptor. Simultaneous addition of only the AT_1_R-ECII (100 nM) or the losartan (300 nM), without AT_1_-AA or Ang II, did not affect the microvascular density when compared with saline control (Figure 4).

## Discussion

These results are the first to demonstrate that AT_1_-AA level is significantly elevated in EOC patients. Enhanced AT_1_-AA titer was associated with advanced stage and grade of the EOC and positively correlated with VEGF level in patients. Using cultured OVCAR3 cells and the CAM of chick embryo, we found that AT_1_-AA has direct effect on cell migration and angiogenesis through activating Ang II AT_1_ receptor.

AT_1_-AA, an autoantibody against angiotensin II type 1 receptor, which is characterized to activate the receptor via specifically interacting with the second extracellular loop of the Ang II AT_1_ receptor, has been documented to play a role in the pathogenesis of preeclampsia and hypertension [10,11,13-15]. However, AT_1_-AA level and function has not been examined or identified in the ovarian cancer. In the current study, we found that serum titer and positive rate of AT_1_-AA were significantly increased in EOC patients. More importantly, this study revealed that the level of AT_1_-AA is significantly elevated with an advanced FGIO stage and grade in EOC patients, supporting the concept that AT_1_-AA may participate in ovarian cancer development and progression. As it has well been demonstrated, the FIGO stage and grade are poor prognostic factors for overall survival in EOC patients [3]. Therefore, monitoring serum AT_1_-AA level might be of great value as a single marker in detecting all stages of EOC patients for clinical screening test, diagnosis and prognosis after therapeutic intervention.

VEGF is a main angiogenic factor in development of ovarian cancer through promoting angiogenesis and significantly associated with tumor progression and poor prognosis [16-18]. Recent studies have shown that targeting inhibition of tumor angiogenesis through VEGF and its various signaling pathways is an effective therapy to suppress tumor growth and progression [8,17]. Our results showed that higher AT_1_-AA titer is positively correlated with VEGF level in advanced stages of EOC patients, consistent with previous findings showing a role of Ang II in cancer development through VEGF gene expression and secretion [8,17].

Stimulation of AT_1_ receptor by Ang II has been reported to be involved in tumor progression in a number of cancers including EOC [12,13]. The postulated role of AT_1_-AA in cell migration and tumor spread led us to test if AT_1_-AA has direct stimulating effect on ovarian cell migration. We selected either autoantibody-neutralizing AT_1_-AA peptide, AT_1_R-ECII as an inhibitor or selective AT_1_ receptor antagonist, losartan to test the direct effect of AT_1_-AA on cell migration and illustrate if this process is mediated by AT_1_ receptor [11]. We found that the migratory number of OVCAR3 cells was significantly increased in AT_1_-AA treated group, which was blocked either by AT_1_R-ECII or losartan. These data suggested that AT_1_-AA has direct effect on migration of ovarian cancer cells through activating AT_1_ receptor, consistent with a previous report showing that Ang II-induced tumor cell invasion, angiogenesis and peritoneal dissemination are blocked by Ang II AT_1_-receptor antagonist [19]. However, mechanistic studies are needed to further elucidate how AT_1_-AA activates the Ang II AT_1_ receptor. In line with our data, it has previously postulated that AT_1_-AA may alter the structural conformation of Ang II AT_1_ receptor so that the receptor’s ability binding to circulating Ang II is enhanced [12].

The CAM of chick embryo has widely been selected to study the morphological aspects of tumor angiogenesis and metastasis [20]. We chose the CAM of chick embryo as a test model to demonstrate angiogenic substances in our study because of its extensive vascularization and easy accessibility to investigate mechanisms of action of proangiogenic and antiangiogenic molecules [20]. We found that addition of AT_1_-AA at the same dose that causes OVCAR3 cell migration is effective in stimulating angiogenesis in the CAM, which was parallel with data showing elevation of VEGF in EOC patients. This increased microvascular density elicited by AT_1_-AA was comparable to the level as that in the Ang II group. Furthermore, we showed that the use of AT_1_R-ECII or AT_1_ receptor blocker, losartan completely inhibits AT_1_-AA-induced angiogenesis of the CAM. These findings suggest that an enhancement of angiogenesis by AT_1_-AA involves activation of Ang II AT_1_ receptor, thus selective Ang II AT_1_ blockade therapy could efficiently inhibit the AT_1_-AA-elicited angiogenesis under conditions exposed to AT_1_-AA as it has previously been reported [19].

There are several limitations to this study that need to be mentioned. First, although *in vitro* studies speculated the mechanisms responsible for the migration of cancer cells and angiogenesis through AT_1_ receptor, this study did not measure AT_1_ receptor expression to show whether such a change is associated with AT_1_-AA-mediated effects. Second, although a raised titer of AT_1_-AA was detected in EOC patients, the “cause-effect” relationship remains to be investigated. In this regard, it will be interesting to determine whether the AT_1_-AA titer falls in patients undergoing treatment. Third, the size of the study population was relatively small and limited only in the Asian patients. Therefore, future large-scale clinical trials will be necessary to further determine whether AT_1_-AA titer is also altered in EOC patients of different ethnicities.

## Conclusions

In summary, we found that serum AT_1_-AA is elevated in higher proportion of EOC patients, which is associated with advanced stages and pathological grades of EOC, and appears to promote the ovarian call migration and angiogenesis through Ang II AT_1_ receptor. This study provides promising data showing that AT_1_-AA may play a significant role in development and progression of EOC, and might be considered as a potential therapeutic target in treatment of EOC patients.

## Abbreviations

AT1-AA: Agonistic autoantibodies against type-1 angiotensin-II receptor; Ang II: Angiotensin II; AT1: Angiotensin II type 1 receptor; AT2: Angiotensin II type II receptor; CAM: Chick embryo chorioallantoic membrane; EOC: Epithelial ovarian cancer; FIGO: International Federation of Gynecology and Obstetrics; OVCAR3: Human ovarian cancer cells; VEGF: Vascular endothelial growth factor.

## Competing interests

The authors declare that there is no conflict of interest that would prejudice the impartiality of this research work.

## Authors’ contributions

LS and HRL participated in research design, patient’s investigation and manuscript writing. JY and SLZ carried out the in vitro experiments and data acquisition; HYX and TL performed data analysis and interpretation. All authors read and approved the final manuscript.
